# Supra-Aortic Vessel Anastomosis in Arch Reconstruction: An Overview of Existing Techniques and Report of a Novel Anastomotic Device

**DOI:** 10.1016/j.atssr.2025.03.018

**Published:** 2025-04-11

**Authors:** Jonathan J. Szeto, John J. Kelly, Lily Huang, David Rekhtman, Jason J. Han, Chase R. Brown, Asad A. Usman, Wilson Y. Szeto

**Affiliations:** 1 Perelman School of Medicine, University of Pennsylvania, Philadelphia, Pennsylvania; 2 Division of Cardiovascular Surgery, University of Pennsylvania, Philadelphia, Pennsylvania; 3 Department of Anesthesiology and Critical Care, University of Pennsylvania, Philadelphia, Pennsylvania

## Abstract

**Purpose:**

This article reviews techniques of supra-aortic anastomosis and presents the use of the novel Duett Vascular Graft System during a complex total arch repair.

**Description:**

The literature was reviewed for existing techniques and devices pioneered to facilitate anastomoses of the supra-aortic vessels during a total arch procedure. A case was then presented to showcase the utility of the Duett Vascular Graft System.

**Evaluation:**

Improving the efficiency of supra-aortic branch vessel anastomosis can reduce circulatory arrest time and the resulting cerebral, spinal, and distal organ ischemia. Therefore, various techniques and technologies have been developed for more expeditious revascularization. The Duett Vascular Graft System is one such device currently in clinical trial that facilitates supra-aortic vessel anastomosis during open aortic arch surgery.

**Conclusions:**

This case showcases the utility of the Duett Vascular Graft System. This novel device allows surgeons to more efficiently perform supra-aortic anastomoses, which can reduce cerebral ischemia and circulatory arrest.

## Technology

The rapid development of novel technologies has allowed cardiac surgeons to successfully treat more advanced aortic arch diseases despite the anatomic challenges of the aorta. However, the arch vessel anastomosis performed in complex aortic arch procedures has not seen similar advancements. The quality of supra-aortic arch vessel anastomosis, as assessed by long-term patency and lack of complications or reintervention, can be the ultimate determinant of the success of an aortic arch procedure.^1^ Currently, the standard method for vascular anastomosis is still conventional suturing. Manual suturing, however, can be time-consuming and technically challenging, especially given the inherent difficulties of performing supra-aortic anastomoses in the aortic arch. These may lead to prolonged brain ischemia or vascular complications, such as thrombosis, stenosis, or bleeding, which contribute to the high mortality and morbidity associated with complex aortic arch procedures.^2^

Various techniques have been developed to more quickly revascularize the supra-aortic vessels with off-the-shelf devices and techniques not specifically designed for arch vessel anastomoses. The Viabahn (Gore Medical) open rebranching technique (VORTEC) is a debranching, telescoping anastomosis that allows branch revascularization with minimal vessel dissection and manipulation.^3^ After minimal surgical dissection of the origin of the target vessel, a guidewire is introduced from the aortic graft into the target artery, and then the Viabahn stent is introduced 2 cm into the target head vessel. The Viabahn device is then deployed and fully expanded by balloon inflation to facilitate the anastomosis between the native head vessel and aortic surgical graft before being secured by transparietal 6-0 sutures.^3^

Modification of the frozen elephant trunk (FET) procedure has also led to new anastomosis techniques. Roselli and colleagues^4^ at the Cleveland Clinic developed the branch stented anastomosis FET repair (B-SAFER) to shorten circulatory arrest time and to reduce bleeding complications by more easily creating a junction between the FET and supra-aortic vessels. In this technique, an aortic endograft is placed antegrade into the descending aorta and modified by creating fenestration in the fabric of the endograft at the orifice of 1 or more of the arch branch vessels, which are still attached to the native aorta tissue. A stent is then delivered directly through the graft fenestrations into the target head vessel over a wire and deployed into the target vessel. The branch stent is then manually dilated with a balloon catheter. Hashizume and colleagues^5^ at Keio University have expanded on this concept with the extended branch stented anastomosis FET repair (EB-SAFER), in which the aorta is transected in zone 1 and the FET is deployed and fenestrated with branch stent grafts in the left subclavian artery (LSCA) and left common carotid artery (LCCA).

Last, supra-aortic vessel anastomosis stent bridging (SAVSTEB) is a modification of the FET procedure that directly connects the aortic graft branch with the corresponding supra-aortic native vessel.^6^ In this technique, the native artery and graft branches are trimmed and then roughly aligned with 2 to 4 interrupted 4-0 Prolene sutures. A stent graft, such as the Viabahn endoprosthesis, is then inserted with a J-wire through the aortic graft branch into the recipient native vessel before being deployed, thus creating the anastomosis.

In this report (Video), we present the deployment of the Duett Vascular Graft System (Aquedeon Medical). The Duett Vascular Graft System, a novel device that has been developed to rapidly connect a native vessel to a polyester surgical graft, is undergoing investigation in an Early Feasibility Study clinical trial (G230197-NCT06253143). This device is a self-expanding covered connector based on an endoluminal, open surgical deployment of a nitinol-reinforced polytetrafluorethylene connection.^1^^,^^7^

## Technique

**Figure 1:**
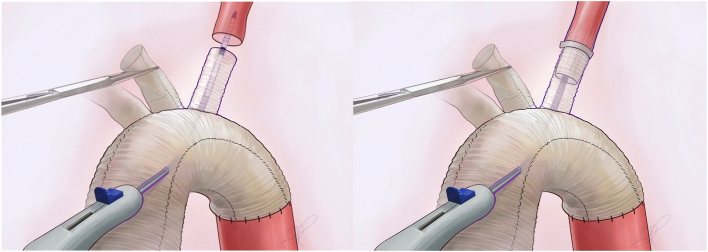
Medical illustration showcasing deployment of the Duett Vascular Graft System in the left subclavian artery.

Total arch (zone 3) reconstruction with a FET is performed with the Thoraflex hybrid plexus device (Terumo Aortic). After trimming of the native supra-aortic artery and corresponding branch of the Thoraflex hybrid device, the Duett system is inserted through a small incision in the Dacron portion of the Thoraflex hybrid device, advanced through 1 of the branches of the Thoraflex hybrid device, and positioned with the distal end of the Duett device 2 cm into the target head vessel (Figure 1). After proper alignment of the Dacron graft and target blood vessel is ensured, deployment of the device creates an anastomosis by directly connecting the surgical graft and target head vessel. The anastomosis is then secured by placing two 4-0 polypropylene stay sutures through the artery and graft.

## Clinical Experience

### Investigation

**Figure 2:**
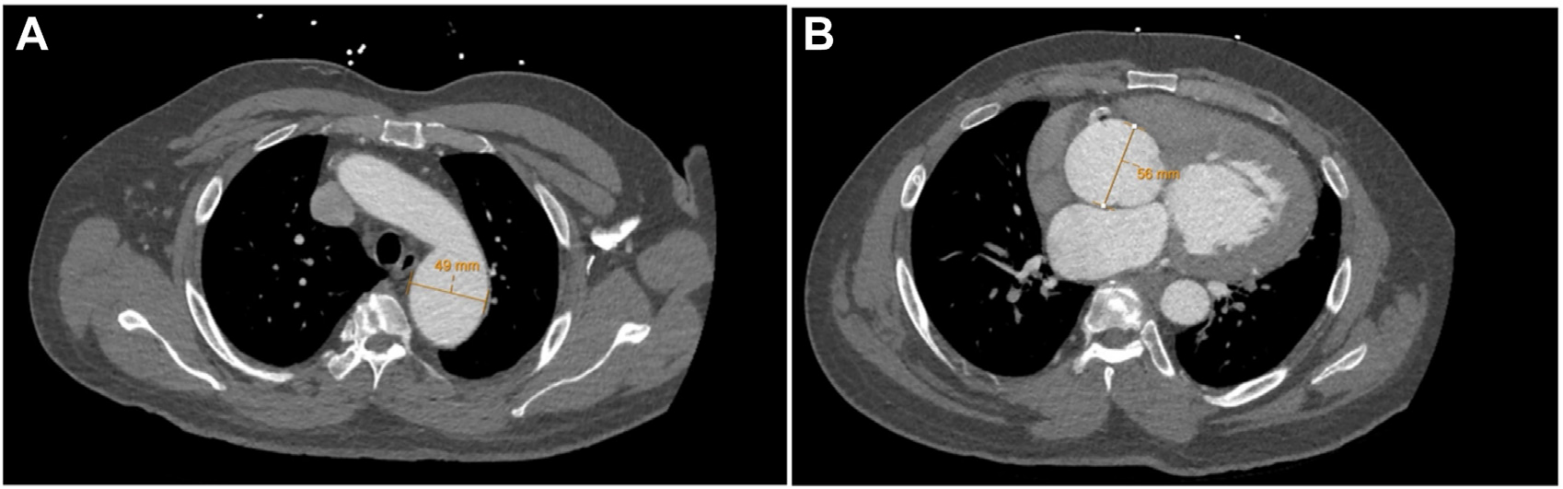
Preoperative contrast-enhanced computed tomography angiography showing measurements of (A) distal arch aneurysm and (B) aortic root aneurysm in axial view.

A 58-year-old man presented to the emergency department for worsening dyspnea on exertion and a past medical history significant for hypertension and hyperlipidemia. Transthoracic echocardiography showed normal left ventricular function but severe aortic insufficiency. Computed tomography angiography showed an aortic root aneurysm measuring 5.6 cm and descending thoracic aortic aneurysm measuring 4.9 cm (Figure 2). Our surgical plan was for elective aortic root replacement and a zone 3 FET total arch reconstruction.

### Operative Technique

After opening of the chest, evaluation of the aortic root confirmed a 5.6-cm aneurysm. The aortic valve was extremely thinned and severely asymmetric, and the noncoronary cusp was tethered. The surgical team opted for a full replacement with no attempt at valve-sparing root replacement. Aortic root replacement with the bioprosthetic Bentall technique was performed.

After cooling to 28 °C, hypothermic circulatory arrest was initiated with bilateral antegrade cerebral protection through right axillary artery cannulation and ostial cannulation of the LCCA. The LSCA, LCCA, and innominate artery were then dissected out, and the distal arch was prepared for a zone 3 anastomosis. A Thoraflex hybrid graft with 36 × 150-mm stent component was deployed into the descending thoracic aorta, and the anastomosis of the device cuff was created in zone 3.

**Figure 3:**
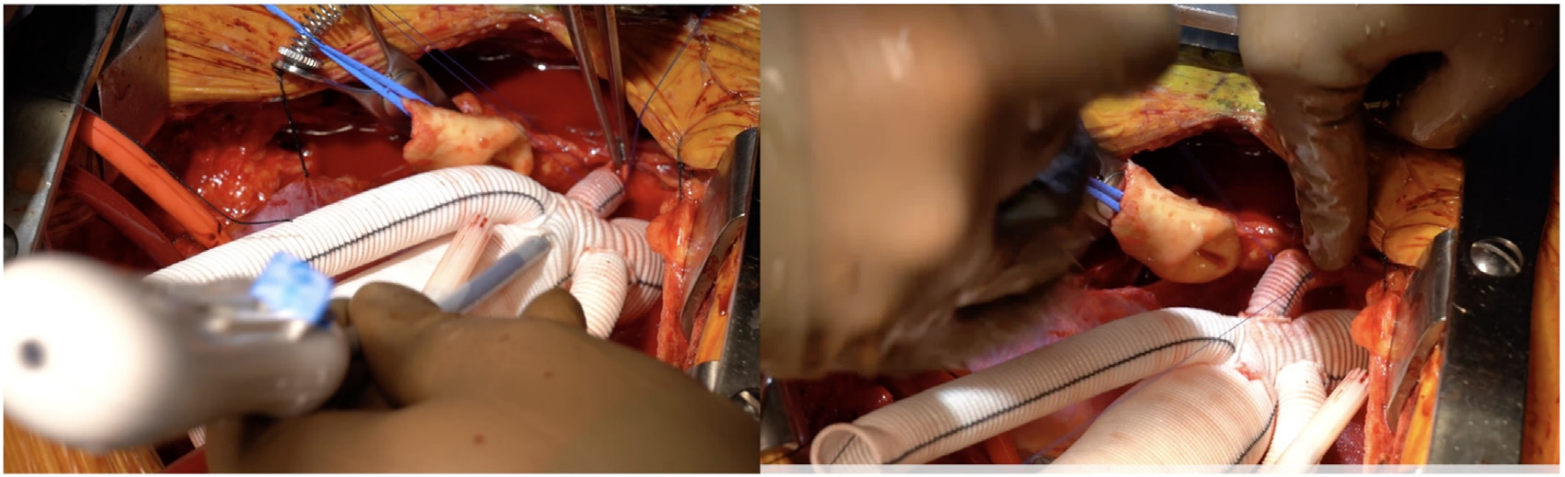
Intraoperative deployment of the Duett Vascular Graft System in the left subclavian artery.

The Duett Vascular Graft System was used for the LSCA and LCCA anastomoses. A 12-mm Duett device was deployed within the native LSCA and the LSCA limb of the Thoraflex device (Figure 3). Next, the LCCA anastomosis was performed in the same fashion with a Duett 10-mm device. Hypothermic circulatory arrest was terminated and cardiopulmonary bypass was reinstituted through the right axillary artery and the perfusion limb of the Thoraflex device. Finally, the innominate artery anastomosis was performed with a running 4-0 Prolene suture in the standard fashion.

After completion of the anastomosis between the aortic root graft and the ascending component of the Thoraflex graft, the cross-clamp was removed and the patient weaned from bypass.

### Postoperative Course

**Figure 4:**
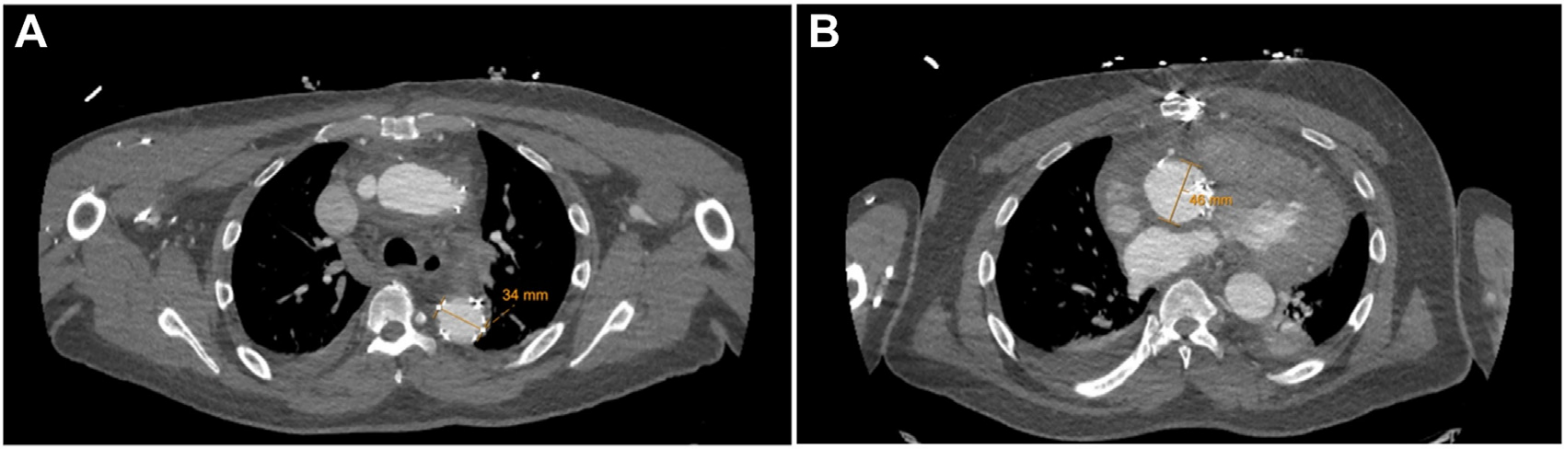
Postoperative contrast-enhanced computed tomography angiography showing measurements of (A) distal arch and (B) aortic root in axial view.

**Figure 5:**
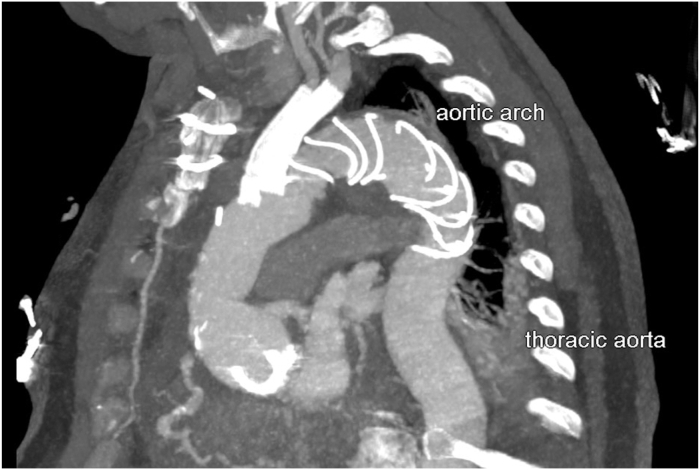
Postoperative contrast-enhanced computed tomography angiography in sagittal view.

There was successful reconstruction of the patient’s root and distal arch, and the patient’s anticoagulation regimen consisted of aspirin and clopidogrel (Figure 4). His postoperative course was complicated by pericardial effusion, for which he underwent pericardial window. Otherwise, his course was uncomplicated, and he was discharged home on postoperative day 9. Excellent flow was noted in both carotid arteries by ultrasound. Predischarge imaging showed patent LCCA and LSCA anastomoses (Figure 5). As of his 6-month follow-up, he continues to walk without limitations, denies any relevant symptoms or complications, and has returned to work.

## Comment

Preserving supra-aortic vessel flow is essential for preventing cerebral and spinal ischemia.^2^ Notably, preserving LSCA flow is especially critical for preserving upper spinal cord collateral blood supply and left arm perfusion. Because of the Duett’s novel design and ease of use, it facilitates the completion of the LSCA vascular anastomosis that can be notoriously both technically difficult and time-consuming, especially during acute dissection or in challenging arch anatomy.

The Duett Vascular Graft System, like the SAVSTEB, creates an orthotopic anastomosis by directly connecting the target supra-aortic vessel to the branch of the surgical graft. As such, the Duett Vascular Graft System has many similar benefits to the SAVSTEB. These include reduced risk of compression and distortion of supra-aortic vessels, and target vessel dissection can be dealt with at the time of the initial operation during acute dissection.^5^ Most notably, when these anastomosis methods are used, the alignment of the surgical graft branches and target vessels is always optimal even if the orientation of the main graft is slightly torqued or the patient’s anatomy is unfavorable. However, the Duett is more intuitive than the SAVSTEB technique as it is specifically designed for anastomosing the supra-aortic vessels. Furthermore, the Duett’s 70-mm-long deployment mechanism allows greater extension deeper into the chest for more challenging anatomy. This was apparent in this case in which the LSCA was deep in the patient’s chest and difficult to access, but an anastomosis was still effectively established.

The Duett establishes an anastomosis between the supra-aortic vessel and the surgical graft directly. This contrasts with the B-SAFER and EB-SAFER techniques, in which the branch stent graft is positioned within a fenestration created in an aortic endograft. Therefore, the anastomosis created by the Duett device is not susceptible to endoleak and aneurysmal expansion, complications seen in cases involving the B-SAFER and EB-SAFER techniques.^4^^,^^5^^,^^8^^,^^9^

However, use of the Duett is limited by available connection sizes. Currently, connectors are made only in 8-mm, 10-mm, and 12-mm-diameter sizes. This restricts Duett’s ability to bridge the anastomosis to the innominate artery, which is usually too large for available connectors. SAVSTEB is similarly limited by Viabahn endoprosthesis sizes (9 mm, 11 mm, 13 mm). The Duett also shares clinical risks with SAVSTEB, such as stenosis, embolisms, stent fractures, occlusions, and bleeding.^6^ However, investigation of the Duett in sheep models showed 100% patency and no thrombosis after 6 months.^10^ Similarly, because the Duett creates an orthotopic anastomotic site instead of a debranching approach, there is less risk of stenosis or distortion compared with the VORTEC technique, which uses long vascular grafts with relatively small diameters.^3^ Last, acutely dissected head vessels are currently a contraindication to this device.

This report showcases the use of the Duett device in a human patient, which successfully facilitated a rapid connection of the Thoraflex hybrid device to the native LCCA and LSCA. We believe that the Duett device is of highest value in patients when the native arch vessels are deep in the chest and manual suturing to create the anastomoses is challenging and time-consuming. Whereas clinical trial of the device is ongoing, the potential reduction in hypothermic circulatory arrest time could reduce the risks of cerebral, spinal, and distal organ ischemia. Moreover, this device can be used by a wide array of surgeons to consistently reproduce anatomic connections and to optimize the quality of arch vessel anastomosis.

## Freedom of Investigation

The tested technology in this manuscript was donated to the study. The authors had full control of the design of the study, methods used, outcome parameters, analysis of data, and production of the written report.

## Disclaimer

The Society of Thoracic Surgeons, The Southern Thoracic Surgical Association, and *The Annals of Thoracic Surgery Short Reports* neither endorse nor discourage the use of the new technology described in this article.
